# Growth Hormone-Secreting Pituitary Macroadenoma: Diagnosis of Acromegaly in a Young Adult

**DOI:** 10.7759/cureus.97829

**Published:** 2025-11-26

**Authors:** Joana Lopo, Ana M Morgado, José Ignacio Moreno, Fatima Cereja, Paula Nogueira

**Affiliations:** 1 Internal Medicine, ULS Algarve - Hospital de Faro, Faro, PRT

**Keywords:** acromegaly, growth hormone, macroadenoma, pituitary hormones, sleep apnea

## Abstract

Acromegaly is a rare endocrinopathy caused by excessive secretion of growth hormone (GH), usually associated with pituitary macroadenomas. Diagnosis is often delayed due to the insidious clinical presentation. We present a 35-year-old male, a baker, previously healthy, who went to the Emergency Department due to episodic, self-limited vertigo. His clinical history revealed chronic night sweats, progressive facial changes, snoring, and breathing pauses. Laboratory tests showed elevated GH and insulin-like growth factor 1 levels (IGF-1), and the MRI confirmed a pituitary macroadenoma. This case illustrates a classic yet underdiagnosed presentation of acromegaly, emphasizing the importance of clinical suspicion even in young and functionally active individuals. Early diagnosis of acromegaly is essential to prevent systemic complications. Recognizing subtle clinical signs can be critical for timely intervention and improving prognosis.

## Introduction

Acromegaly is a chronic and rare endocrine disorder, predominantly caused by GH-secreting pituitary adenomas, leading to persistently elevated IGF-1. Its estimated prevalence ranges from 40 to 70 cases per million people; it affects men and women equally, with most diagnoses occurring in the fourth or fifth decades of life [1]. The slow progression of the disease makes early recognition challenging. Classic signs include facial changes (prognathism, macroglossia), enlargement of extremities, sweating, headache, fatigue, and sleep apnea. Despite being typical, these findings are often attributed to ageing or lifestyle factors, resulting in an average diagnostic delay of seven to eight years [2].

Growth hormone (GH) and insulin-like growth factor 1 (IGF-1) are central regulators of growth, metabolism, and tissue maintenance, and their physiological balance depends on normal pituitary function. A pituitary macroadenoma, defined as a benign tumor of the pituitary gland measuring more than 10 mm in diameter, can profoundly disrupt this balance by altering GH secretion. When the adenoma arises from somatotroph cells, it may lead to GH hypersecretion, resulting in elevated IGF-1 levels and the clinical syndromes of acromegaly in adults or gigantism in children. The excessive GH and IGF-1 stimulate abnormal somatic growth, soft tissue proliferation, insulin resistance, and metabolic dysregulation, contributing to characteristic manifestations such as enlarged extremities, facial bone overgrowth, organomegaly, and cardiovascular complications [2].

Conversely, a nonfunctioning pituitary macroadenoma may compress surrounding pituitary tissue and the pituitary stalk, leading to hypopituitarism and consequently reduced GH and IGF-1 levels, which can manifest as fatigue, increased adiposity, loss of muscle mass, and impaired quality of life. Moreover, macroadenomas may exert mass effects on nearby structures, such as the optic chiasm, causing visual field defects. Therefore, understanding the physiological roles of GH and IGF-1 is essential in interpreting the clinical and metabolic consequences of pituitary macroadenomas and in guiding both diagnostic evaluation and therapeutic management. Conditions that may mimic acromegaly include gigantism, Paget’s disease of bone, hypothyroidism, familial acral enlargement syndromes, and pachydermoperiostosis. Clinical and biochemical differentiation is essential for accurate diagnosis. The diagnostic process involves biochemical confirmation and etiological investigation: serum IGF-1 measurement, oral glucose tolerance test (OGTT), pituitary MRI, and additional studies such as visual field testing and metabolic evaluations.

This article reports the case of a young man with classic but unrecognized symptoms, whose diagnosis of acromegaly was established only after evaluation of nonspecific complaints. Patients usually report nonspecific complaints such as fatigue, sleep disturbances, or joint pain that precede the facial or extremity changes. This insidious course of the disease is why it's frequently misdiagnosed. We discuss the clinical, hormonal, radiological, and therapeutic aspects of the disease.

## Case presentation

A 35-year-old male, baker, previously healthy, in the last six months, presented with episodic, self-limited vertigo, along with chronic night sweats, occasional headache, snoring, and breathing pauses (for at least two months, according to his wife). He also noted progressive enlargement of his hands and feet and changes in facial appearance, such as a broader nose and prominent chin.

**Figure 1:**
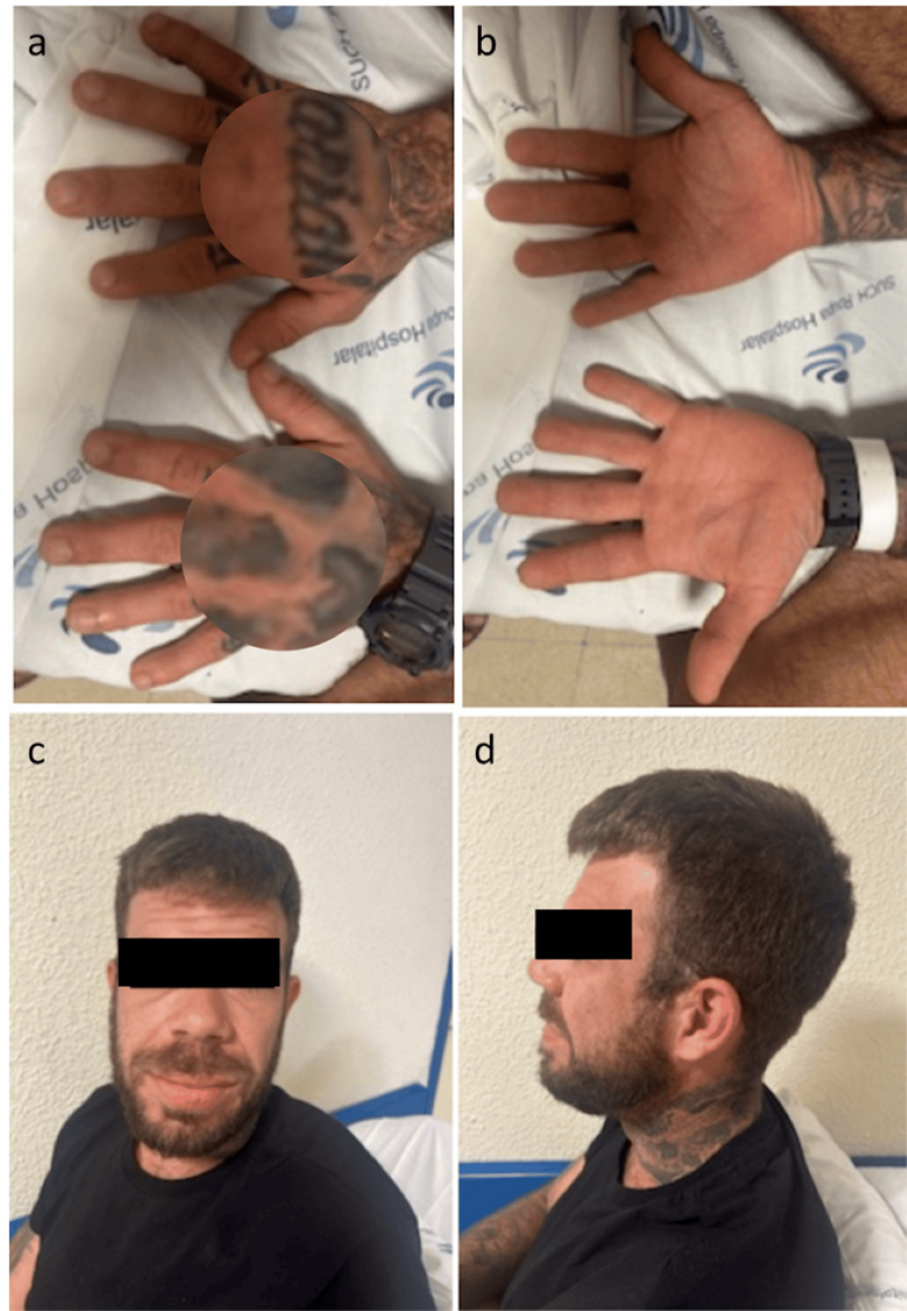
Physical characteristics of acromegaly. (a, b) Images show skin thickening and enlarged hands. (c, d) The patient's facial tissue hypertrophy due to chronic growth hormone excess is evident. Also noted is the enlarged mandible and broadened nasal bridge. (Image published with patient's consent)

During the physical examination, it was possible to notice, as seen in Figure 1, an acromegalic facies, including frontal skull bossing; resulting in an unusually prominent forehead, mandibular prognathism, and skin thickening and enlarged hands and feet. Vital signs were stable, and there were no neurological deficits.

**Figure 2:**
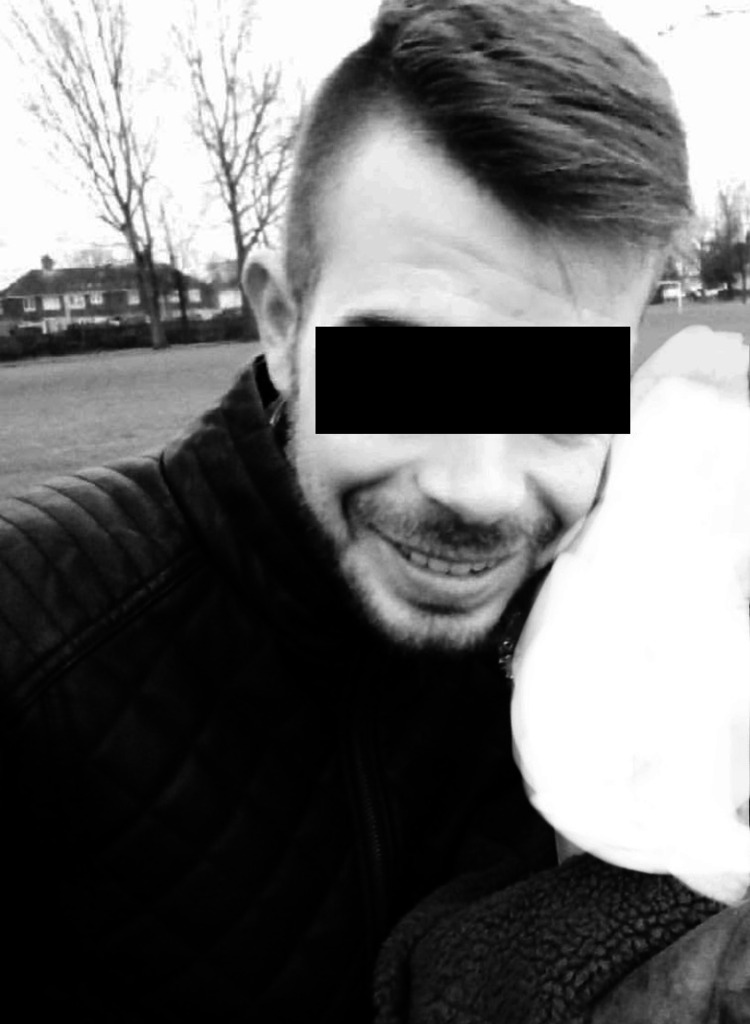
Physical features of the patient in 2020. (Image published with patient's consent).

Facial features of the patient prior to the onset of symptoms (2020), where a thinner skin, a narrower nose, and a slimmer mandible can be observed (Figure 2). 

The hormonal study is described in Table 1. Laboratory tests revealed extremely high GH and IGF-1 levels, confirming acromegaly. No decrease in GH levels after an oral glucose challenge shows that feedback inhibition is lost. 

**Table 1:** Laboratory results (assay methods: chemiluminescence). * No suppression of GH after 75 g oral glucose challenge. GH: Growth hormone, IGF-1: Insulin-like growth factor 1, ACTH: Adrenocorticotropic hormone.

Hormone	Value	Reference
GH	32.6 ng/mL	0.06-5 ng/mL
GH* (Oral glucose tolerance test)	32.4 ng/mL	*post-glucose GH value
IGF-1	473 ng/mL	81-222 ng/mL
ACTH	28 pg/mL	7.2-63.3 pg/mL
Morning cortisol	9.8 µg/dL	7-21 µg/dL
Total testosterone	459 ng/dL	253-803 ng/dL

**Figure 3:**
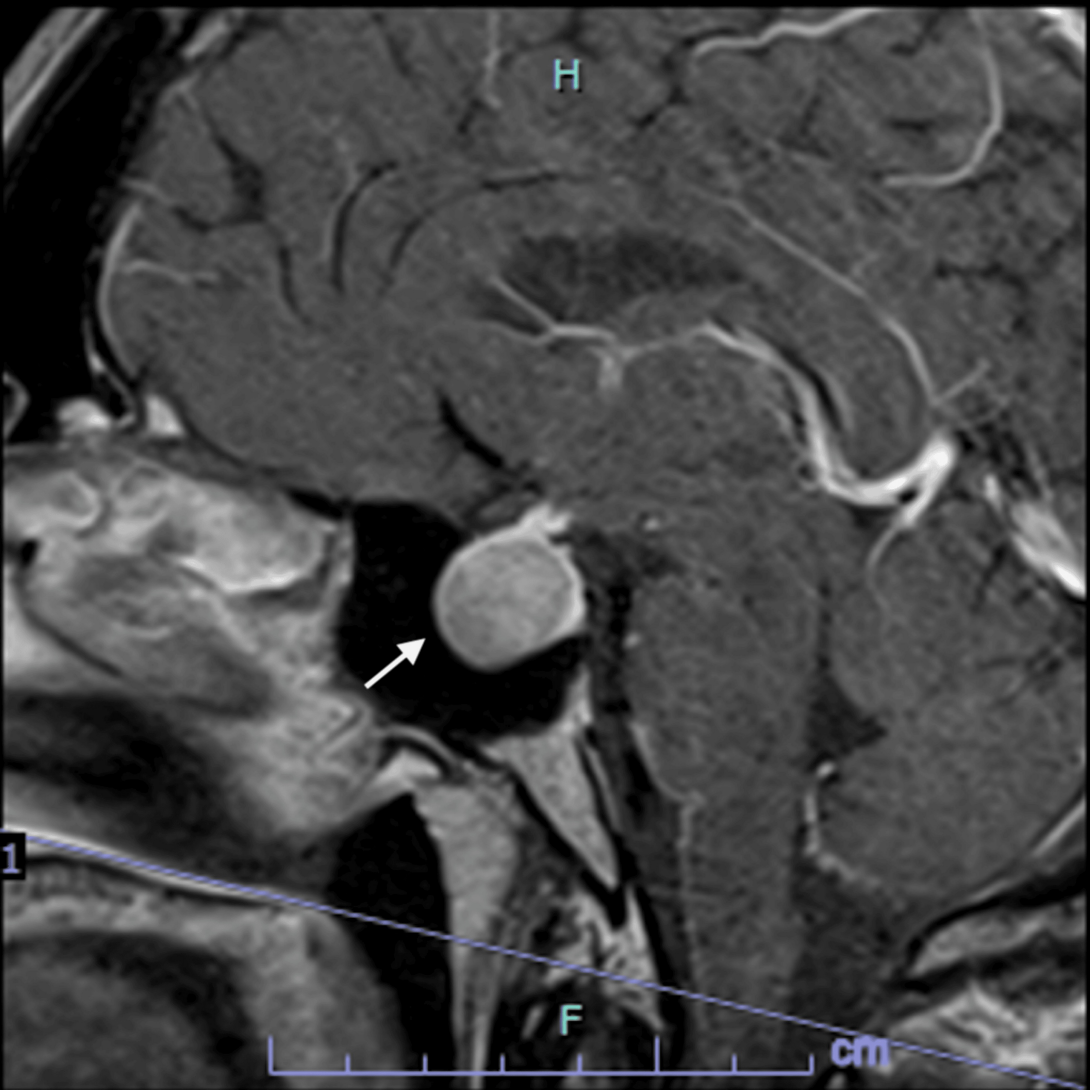
MRI Sella turcica. MRI of the Sella turcica (Coronal T1-weighted image after gadolinium ‍‌‍‍‌‍‌‍‍‌contrast‌‍‍‌‍‌‍‍‌) showing a 17×23×17 mm pituitary macroadenoma extending superiorly to compress the optic chiasm (arrow).

In the MRI of the Sella turcica was possible to confirme an expansive intra- and suprasellar lesion, isointense relative to the cerebral cortex on T1 and T2, represented by a focal area of low uptake after administration of paramagnetic contrast, measuring approximately 17 x 23 x 17 mm (craniocaudal, transverse, and anteroposterior diameters, respectively), which compresses the optic chiasm and obliterates the suprasellar cistern in a possible relationship with a pituitary macroadenoma (Figure 3). Because the patient did not have visual symptoms, but he could not explain more about the dizziness, a perimetry was ordered to clarify this subject. 

## Discussion

Acromegaly is a rare and slowly progressive condition, resulting from chronic exposure to excess GH and, consequently, IGF-1, leading to somatic, metabolic, and cardiovascular alterations [1]. It is estimated that the average time between the onset of symptoms and diagnosis is approximately 7 to 8 years, highlighting the insidious nature of the disease and the low clinical suspicion in its early stages [2].

The patient presented with classic signs such as acral changes, acromegalic facies, snoring and breathing pauses, often overlooked for years. Sweating, although nonspecific, is a symptom reported in up to 70% of patients [3]. The initial complaint of dizziness may have prompted the patient to seek medical attention, though it is not a classic symptom of acromegaly. There are occasional reports of dizziness or symptoms suggestive of autonomic dysfunction in acromegalic patients, although the exact prevalence of this symptom is not yet well established.

Laboratory tests showed elevated serum GH (32.6 ng/mL) and IGF-1 (473 ng/mL) levels, as well as a lack of GH suppression after glucose administration, confirming the diagnosis of acromegaly. The patient's IGF-1 level was approximately twice the upper limit of normal, which aligns with the moderate clinical severity and macroadenoma size noted. Sella turcica MRI revealed a pituitary macroadenoma, consistent with most cases; approximately 71% of GH-secreting adenomas are macroadenomas (>10 mm) at the time of diagnosis [4].

Another relevant aspect in this case is the partial preservation of pituitary function, with ACTH and testosterone levels within normal limits, and morning cortisol at the lower limit of normal. In many patients with macroadenomas, compression of normal pituitary tissue may occur, leading to hypopituitarism, which had not yet manifested clinically in this patient. Regular endocrine monitoring is essential, even after surgical treatment, due to the possibility of hormonal axis deterioration or partial recovery.

From a systemic perspective, acromegaly is associated with several complications, including hypertension, acromegalic cardiomyopathy, diabetes mellitus, obstructive sleep apnea, joint disorders, and an increased risk of colorectal neoplasms [5]. In the presented case, there were already indications of obstructive sleep apnea, snoring and sleeping pauses, a common and underdiagnosed manifestation that can significantly impact morbidity and mortality.

The treatment of choice, especially in cases with macroadenomas and no invasion of critical structures, is transsphenoidal surgery. The goal is complete tumor resection, hormonal normalization, and relief of local compressive symptoms. However, only 40% to 50% of patients with macroadenomas achieve biochemical remission after surgery alone. Medical therapy, such as somatostatin analogues, GH receptor antagonists, and dopamine agonists, is indicated in cases not cured by surgery, and radiotherapy remains an option for aggressive or residual tumors [6]. In this case, the patient was referred to the speciality of endocrinology, who asked for a perimetry to clarify the dizziness before the beginning of medical treatment. According to the response of patient, surgery will be considered. The disease staging is still in progress. 

## Conclusions

Acromegaly is a chronic, progressive, and underdiagnosed disease with an insidious course, in which clinical signs are often overlooked. The recognition by the authors of the clinical signs, such as the alteration in the bone structure and body size, was very important to increase the suspicion level. This case highlights not only the importance of physical examination and focused clinical history but also the crucial role of clinical suspicion in young patients with chronic and seemingly benign symptoms. The combination of these factors, along with appropriate hormonal assessment, was essential for an accurate diagnosis. Although rare, acromegaly is a treatable disease. Early detection cannot only improve quality of life but also reduce comorbidities and increase patient survival. This clinical case is relevant as it demonstrates that acromegaly may remain silent for many years, and that attention to clinical details is key to avoiding diagnostic delays and initiating treatment before irreversible complications develop.
